# The Etiology and Treatment of Typhoid Fever

**Published:** 1861-06

**Authors:** V. H. Taliaferro

**Affiliations:** Columbus, Georgia


### ARTICLE II.

*The Etiology and Treatment of Typhoid Fever.* By V. H. TALIAFERRO, M. D., of Columbus, Georgia.

Read before the Medical Association of the State of Georgia, April, 1861.

CONCLUDED.

### THE BRAIN AND NERVOUS SYSTEM.

There is no lesion of the brain peculiar to typhoid fever, and no *relation* between the cerebral symptoms, and the pathological appearances. From the well marked and almost constant cerebral disturbances, we would naturally be led, aside from the revelations of pathology, to expect distinct and serious lesions of the brain, or its coverings. Far from this, the brain is usually found in its normal state. Where, then, are we to look for a solution of these disturbances, and the almost invariable *death by coma?* To no other source can we go but to the blood for a poison, which has made its specific impression upon the great vital functions of the brain and nervous system. Prof. Watson says: "But the exciting cause in most cases, probably in all, is a *specific poison* introduced into the blood. The first evidence of the circulation of this altered fluid is *depression of the powers and functions of animal life.*" This poison is clearly, in my humble judgment, received into the blood, from a disturbance of the *depurating functions* of the cutaneous system, whereby excrementitious and noxious matter has been retained in the circulation. Indeed, where else are we to find a poison of sufficiently general distribution, calculated to effect such *overwhelming results* as characterize typhoid fever? Certainly not from a specific contagion, for the disease is known by almost every practitioner in America to exist independent of any contagious principle. Certainly not from *inflammation* of the mucus membrane of the small intestines, or the glands of Peyer, for the phenomena of typhoid fever have no relation with the results and products of inflammation. Prof. Jackson says: "But the *softness* which has been noticed in so many organs is thought by the best pathologists not to be inflammatory, but rather the result of a direct loss of *vital cohesion* in the organs, either from debility, or the *state of the blood.*"

In obscure diseases, or where their immediate causes are involved in obscurity, the *mode of death* by which the subject dies, becomes a matter of vast importance. This fact was particularly inculcated by Cullen, the better thereby to "obviate the tendency to death." In typhoid fever the usual mode of death is unquestionably *by coma.* Now, by what causes are coma produced? Watson says: "coma may result from at least two different kinds of cause. One cause is pressure, which is mechanical." This cause, we very well know, has no connection with the production of the coma of typhoid fever. "Another, which is probably chemical, is the circulation of some noxious or narcotic substance in the blood." Furthermore, continues Prof. Watson: "Fatal coma, without obvious disease in the brain to account for it, results not unfrequently *from an inbred poison*, of which the agency was not recognized until a recent period: *the poison of unpurified blood.*" Now, will the excrements of the skin so poison the blood as to result in death by coma? Certainly there are none to deny that they will. All depurating or excrementitious matter is noxious and poisonous to the blood, and therefore, if we are to give any credence to the distinguished authority just quoted, is fully capable of causing *coma.* Considering typhoid fever as essentially an *intestinal disease*, death should take place in the way of syncope.

KIDNEYS.

In a recent French work, by Frederic Durian, upon typhoid fever, the kidneys are said to be sometimes inflamed, presenting here and there purulent points.* We have already seen that the kidneys are sometimes profuse in their action, and usually the secretion is above or about the normal standard. This can be accounted for only upon the presumption that the kidneys, acting vicariously with the skin, have been overstimulated in their function of depuration, in the effort to relieve the blood of its foreign and excrementitious elements.

---

We are indebted to Dr. B. L. Jones, of Montgomery, Ala., for the translation from the French.

### TREATMENT.

*Skin.*—In the treatment of typhoid fever, our attention should first and foremost be directed to the cutaneous system. This will very generally be found incrusted by the collection of the perspiratory fluids and debris of the skin, and imparting to the hand thereby a peculiar dry and unpleasant feel. This condition will not be ascertained by mere inspection, but will require the use of *friction*, *warm water and soap*. Friction should first be applied to loosen and break up the closely and firmly impacted debris. This should be immediately followed by warm water and soap, applied with a large, coarse towel; after which the surface should be thoroughly dried. This should all be done while the patient is lying upon a hard mattrass, overspread with oil cloth, and repeated once in twenty-four hours. Early in the morning, before any nourishment has been taken, is by far the most preferable time. The refreshment and renewed vigor thus afforded is often astonishing.

In addition to the bath, the patient should every night have the *entire surface* well *greased*, using as much friction in the application as comfort will permit. For this purpose, either lard, olive oil, or *bacon rind* may be used, applied always with the *hand* of an attendant. If the lard, or oil, is used, it should be made to contain as much *salt* as it will dissolve. Fresh lard is perhaps preferable. Care should be observed that no *uncleanly* or *rancid* grease is used, as it would prove more *irritating* than soothing.

The reasons for thus annointing typhoid patients are simply these: In the first place, a hard, dry, horny cuticle is made *soft* and *pliant*, its texture loosened, and opened, extraneous matter made readily detached, the reinstatement of its lost functions thereby encouraged, and the nervous system relieved of the irritation necessarily imparted to it by the contact of the sensory nerves with a dry horny cuticle. The salt is added for its antiseptic and disinfectant properties, whereby is obtained a highly salutary and counter influence to the altered and putrescent condition of the blood, and indeed of all the tissues. Not only are its benefits obtained by the absorbant powers of the skin, which carries it directly into the circulating mass, but by its neutralizing powers upon the fœted and cadaveric odors of the body.

*Exercise.*—Another important result to be obtained by this regularly repeated bathing and greasing process, is the *exercise* thereby forced upon the patient. The benefit thus derived is apparent, when we consider that typhoid patients lie upon their backs, if permitted, for days and even weeks, thus favoring congestion of the languid and almost lifeless viscera, and inducing a general torpor, by the continued lifeless inactivity. It is a matter of paramount importance, that we give our patients a sufficiency of *rubbing* and *rolling.*

*Clothing, Bedding, &c.*—Owing to the putrescent character of typhoid fever, *cleanliness* becomes an object in its management, of the first magnitude. There should be an entire change of the patient's clothing and bedding after each morning ablution, that there may be no further accumulations upon the surface, and no reabsorption of vitiated secretions. We should loose no opportunity in giving every encouragement and assistance to the reinstatement of the functions of the skin.

*Air and Sunlight.*—A complete and perfect ventilation of the patient's chamber should never be overlooked. We should be assured that nothing is wanting, in the way of ventilation, to give a perfect oxygenation of the blood. A neglect of this simple precaution may result very seriously to the patient. The blood in this disease, perhaps more than any other, requires the vitalizing influences of oxygen. It is no uncommon practice to admit the barest possible amount of air through the day, and to exclude it entirely at night. The erroneous impression is widely prevalent that night air is highly deleterious to the sick, and if the physician's attention is not especially given to this fact, his patient will frequently be subjected to all the hazardous influences of confined air, which is being breathed and rebreathed by the invalid and his attendants.

Sunlight should be freely admitted from time to time in the patient's chamber, for its mildly stimulant and tonic influences to the circulatory and nervous systems. The very common practice of *darkening* the room is founded upon false ideas, and can have no other result than to still further deprave the already impoverished blood. *A greatly depraved* and *enervated constitution cannot be renovated in the dark.* A fitting example of the influence of sunlight upon the circulating fluids of animals, may be found in the greatly impoverished condition of plants which have grown in the dark: the coloring matter of the fluids being almost entirely wanting.

*Diet.*—The dietetic management of typhoid fever cannot be too closely studied. The long continuance of the disease, as also the rapid disintegration which attends it, demand a most vigilant attention to the necessary repetition of such diet as will rest lightly upon the stomach, and may be easily and thoroughly assimilated. The patient's repugnance to diet is no excuse for its not being given. If in no other way, it should be administered as a medicinal agent. The best preparations, and those I am accustomed to use most frequently, are *beef tea* and *milk punch* alternately. They should be given every two or three hours, as the case may seem to require. I am very well satisfied that hundreds die in this disease for want of sufficient and proper nourishment. We should not wait (as is very common) for the first stages of the disease to pass before beginning a system of nourishment. It should be commenced at once—in the beginning of the disease, and the vitality and energies of the system thereby, as much as possible, sustained.

*Brandy or Wine* may be given in greater or less quantities, as circumstances seem to demand. It should be given in the earlier as well as latter stages of the disease. Sufficient caution should be observed in its administration, for *too much* may be given and the system depressed instead of sustained. I have yet to understand the philosophy of those who withhold such agents until they have the most unmistakable evidences of *departing life.* Brain disturbances, which deter some in the use of brandy, are by no means contraindications to its use, but on the other hand leading indications for its bold administration.

In *coma*, brandy is the great remedy, and indeed the only remedy with which we may confidently hope to wake up the system from its deep and death-like slumbers. Coma, as we have seen, results from a blood-poison and not from a *lesion*, and the impression of *brandy* is stimulant and counter to that poison.

*Chlorate of Potassa.*—The therapeutic properties of this remedy may be reckoned as follows: 1st, It is a blood depurant; 2nd, *antiseptic;* 3d, alterative; 4th, tonic.

Aside from these it seems to have some specific virtue upon ulcerated mucus membranes. "It increases the acidity of the urine, and is said by Isambert to cause a more free deposit of urates and pigments." [Edmund A. Parkes, M. D., London.] Thus does it appear that in one remedy is blended all the virtues calculated to fill the more important and essential indications of typhoid fever. Patients submitted to its full influence rarely exhibit that marked *putrescent* condition so common, and so characteristic of the severe forms of the disease; the bowels are far more controllable, and *coma* is rarely, if ever, present.

In its administration, we may safely calculate upon the introduction of a large amount of oxygen into the system, with its stimulant and vitalizing effects upon the depraved and vitiated blood of typhoid fever. Also, we may calculate upon its *certain antiseptic influences* upon the fluids and tissues. To obtain these results is certainly of vast importance, for we have seen that the mucus membrane, spleen and and heart tend to a complete loss of vitality and disorganization, evidenced by the frequent occurrence of *softening* of those parts. To avert this tendency should command our first and most vigilant attention.

*Veratrum Viride.*—This remedy may be combined with the potassa as a sedative to the circulation, as the indications arise for its use. If there be very great excitement of the heart, the veratrum, as the most efficient, safe and certain sedative to that organ, should be given; caution in the meantime being observed against nausea and vomiting, with their depressing results. It is an easy matter, with a little care, to produce a sufficient sedation with veratrum, without nausea or the least vital depression.

*Opium.*—This will be needed for two purposes, viz: to quiet nervous disturbance and insure sleep at night, and to control the action of the intestines. It should be used as these indications arise. Cerebral disturbances should by no means deter us in the use of opium. No better remedy can be administered for the *delirium* of typhoid fever. The best time for its administration is at night, when a decided dose should be given, of *pure opium*, and the patient left undisturbed to his repose, until the next morning. The practice of continually arousing the patient through the night for the purpose of physicing him is a hazardous one.

*Purgatives.*—This class of remedies should be wholly ignored. They are inappropriate at any stage of the disease. The bowels may be constipated, but a purgative, while unloading the intestines, may rapidly and actively develop the disease to which they so greatly predispose. Where it is necessary to unload the bowels, cold water enema should be used. If this does not suffice, warm water, with a little soap or meal, may be used. After the bowels have been once sufficiently moved by these means, no other laxative should be employed save *cold water enema.* While, by this means, a sufficiently laxative effect is obtained, we also make a decided *tonic* impression upon the mucus membrane of the intestines. Dr. J. S. Wilson, of Columbus, Ga., who has contributed to the profession much valuable and interesting matter upon the subject of Hydro-Therapeutics, says: "Cold water enemata in typhoid fever are particularly serviceable in that distressing tympanetic condition of the bowels, so commonly found; not alone for their tonic and sedative properties, but for their power of *condensing* the confined gasses." Certain it is, that their effects are highly salutary, and should not be overlooked. In connection with the cold water enemata the external application of cold water to the abdomen, by means of the sponge or cloths, will be found highly serviceable in that *tense*, *hot* condition of the bowels which so frequently proves perplexing and annoying to the physician, as well as the patient. The cold should not be applied longer than required to subdue the heat. According to Dr. Burney, in the Southern Medical and Surgical Journal, great caution should be observed in the use of cold, wet clothes. He says: "To attain this end, let towels be folded, dipped in cold water, and laid over the abdomen, removing every ten or fifteen minutes, till the heat is overcome. Nothing exerts a more potent influence over the circulation, and we have seen the patient collapsed, in this very way, in a few hours; hence it must be evident to all, the remedy is not admissible, provided the attack is of long standing, and even when properly used must be closely watched—the attending physician using it himself, narrowly watching the pulse, and always stopping it as soon as the temperature of the skin over the bowels is properly reduced."

### THEORIES AND THERAPEUTICS OF TYPHOID FEVER.

We now propose to review, very briefly, some of the most prominent theories of typhoid fever, with the plans of treatment as based upon most theories.

The theory, originating with the French pathologists, which makes typhoid fever a *follicular enteritis*, or a local phlegmasia, is the one now most generally assumed in Europe and in America. The treatment based upon this theory should be, of course, *antiphlogistic and depletent*, embracing venesection, mercury, antimony, etc. Does experience and observation teach us that this is the most correct and *successful* plan of treatment? Could the many who have been unceremoniously hastened to eternity give answer, we would doubtless hear NO, echoed and reëchoed from the hills and the valley—from the mountains to the sea-board.

Another theory is that in which the nervous system becomes the primary seat of disorder. Prof. Jno. G. Westmoreland, who assumes this theory, locates the primary and essential lesions in the *madulla* oblongata. As his views are novel, we present them in his own words: "Fevers called idiopathic, such as typhoid, typhus, intermittent and remittent, and yellow fever, are dependent upon a poisonous influence exerted upon some portion of the nervous system directly, as a specific action; and the poison leading to each of these fevers is a special one, effecting a particular portion of the nervous system specifically. These distinct impressions lead to symptoms common to all: such as fever and functional and organic disturbance, while in others, such as time of duration, adynamic tendencies and particular local symptoms, they differ materially. The poisoning is effected by agents, the character and precise origin of which are not well understood. Their action is not such as usually produces permanent organic lesion of the part affected, but disturbs the functions always. It is no more a stretch of the imagination to conclude that different portions of the nervous system are selected by these separate poisonous influences, than it is to believe the fact that opium selects the brain for its action, whilst strychnine, with equal uniformity, effects the spinal marrow. We know these parts are impressed by the drugs named, from the invariable symptoms which follow their administration. Some symptoms common to both may be found, yet there are special evidences of disturbance in a particular part of the nervous system. Now we have in the particular influence or poison which produces the symptoms characteristic of typhoid fever, a particular property by which it selects, in the same way, a particular portion of the nervous centers, the madulla oblongata, if you please, upon which its action is exerted. From this we have some symptoms in common with simular disturbance of the middle and lower portion of the madulla spinalis, and others which differ widely. Arising from the former, we would expect symptoms of more general and perfect depression of the vital or nervous influences in the animal economy, than in the latter; and in addition to the difference in general symptoms, local evidences, such as pains, tenderness, &c., direct attention to the point of primary disturbance. Then we conclude, that as malarial, spinal or remittent fever, gives general and local evidences of disturbance primarily in the middle and lower portions of the spinal cord, it is upon this part the poison of malaria exerts its action; and on the other hand, as the evidences of disturbance in the madulla in typhoid fever are equally conclusive, we feel warranted in attributing its origin to that cause. The peculiar local disease found in the bronchia, bowels and parotid glands, in typhoid fever, can be accounted for in the fact of deficient nervous influence affecting the capillaries of these parts."

Admitting this theory, what, we would ask, should be the most plausible and philosophic plan of treatment? It is to *treatment* that all theories culminate at last, and aside from this they amount to naught. Taking then this theory as a basis, those therapeutic agents should be most efficient which act *directly* upon the nervous centers, creating a counter or alterative effect, such as quinine, strychnine, &c. Now we believe that it is a very generally conceded fact, that quinine is wholly inefficient in typhoid fever, and in the opinion of many, in which I fully coincide, actually hurtful. The Doctor's treatment, far from embracing such agents, I believe consists mainly in *soup* and *brandy*, by the way a most excellent treatment.

"Another theory is that which places the primary and fundamental changes in the blood." It is indeed here that the morbid phenomena of the disease would seem to take their origin, but we cannot of course pretend to say whether the poison, or noxious excrements, which have been described, make their impression first upon the nervous system or blood. Regarding the blood, however, in the light of an organized *vital fluid*, we cannot well see how noxious or poisonous agents introduced into it, could result otherwise than in a *direct* morbid impression. The advocates of this blood theory admit the existence of a poisonous agent, of which they have no knowledge. Watson says it depends upon a specific poison introduced into the blood, and "all analogy is in favor of the belief that the primary change is wrought in the blood" "The whole mass of the blood is gradually vitiated; and the first evidence of the circulation of this altered fluid, is depression of the powers and functions of animal life." Upon this theory a highly valuable and efficient system of treatment has been founded, consisting of depurating and sustaining agents. The treatment of typhoid fever, however, has been largely empirical—remedies being used regardless of any principle in their application.

These theories, with their attendent therapeutics, as compared with the *cutaneous theory*, as herein set forth, with its plan of treatment, are most respectfully and humbly submitted.

